# P-glycoprotein Mediates Resistance to the Anaplastic Lymphoma Kinase Inhiitor Ensartinib in Cancer Cells

**DOI:** 10.3390/cancers14092341

**Published:** 2022-05-09

**Authors:** Chung-Pu Wu, Cheng-Yu Hung, Megumi Murakami, Yu-Shan Wu, Chun-Ling Lin, Yang-Hui Huang, Tai-Ho Hung, Jau-Song Yu, Suresh V. Ambudkar

**Affiliations:** 1 Graduate Institute of Biomedical Sciences, College of Medicine, Chang Gung University, Taoyuan 33302, Taiwan; chunling1019@gate.sinica.edu.tw (C.-L.L.); d000014064@cgu.edu.tw (Y.-H.H.); yusong@mail.cgu.edu.tw (J.-S.Y.); 2 Department of Physiology and Pharmacology, College of Medicine, Chang Gung University, Taoyuan 33302, Taiwan; 3 Molecular Medicine Research Center, College of Medicine, Chang Gung University, Taoyuan 33302, Taiwan; aruhung@mail.cgu.edu.tw; 4 Department of Obstetrics and Gynecology, Taipei Chang Gung Memorial Hospital, Taipei 10507, Taiwan; thh20@adm.cgmh.org.tw; 5 Laboratory of Cell Biology, Center for Cancer Research, National Cancer Institute, NIH, Bethesda, MD 20892, USA; megumi.murakami@nih.gov (M.M.); ambudkar@mail.nih.gov (S.V.A.); 6 Department of Chemistry, Tunghai University, Taichung 40704, Taiwan; yushanwu@thu.edu.tw; 7 Department of Medicine, College of Medicine, Chang Gung University, Taoyuan 33302, Taiwan; 8 Department of Obstetrics and Gynecology, Keelung Chang Gung Memorial Hospital, Keelung 204, Taiwan; 9 Department of Biochemistry and Molecular Biology, College of Medicine, Chang Gung University, Taoyuan 33302, Taiwan; 10 Liver Research Center, Linkou Chang Gung Memorial Hospital, Taoyuan 33302, Taiwan

**Keywords:** P-glycoprotein, multidrug resistance, ALK, ensartinib, X-396

## Abstract

**Simple Summary:**

P-glycoprotein (P-gp; ABCB1) is the most well-characterized ATP-binding cassette (ABC) multidrug efflux transporter known to actively transport a wide variety of cytotoxic and molecularly targeted drugs out of cancer cells, causing multidrug resistance (MDR) and poor prognosis in cancer patients. In this study, we investigate whether P-gp overexpression can contribute to reduced susceptibility of cancer cells to the anaplastic lymphoma kinase (ALK) inhibitor ensartinib. We discovered that P-gp-overexpressing cancer cells and cells with ectopic expression of P-gp are significantly less sensitive to ensartinib than the respective parental cells. By blocking the drug transport activity of P-gp, the intracellular accumulation and cytotoxic activity of ensartinib were fully restored in P-gp-overexpressing cancer cells. Moreover, in silico molecular docking analysis of ensartinib with the inward-open structure of P-gp provided additional information on the potential binding orientation of ensartinib in the substrate-binding pocket of P-gp.

**Abstract:**

Ensartinib (X-396) is a promising second-generation small-molecule inhibitor of anaplastic lymphoma kinase (ALK) that was developed for the treatment of ALK-positive non-small-cell lung cancer. Preclinical and clinical trial results for ensartinib showed superior efficacy and a favorable safety profile compared to the first-generation ALK inhibitors that have been approved by the U.S. Food and Drug Administration. Although the potential mechanisms of acquired resistance to ensartinib have not been reported, the inevitable emergence of resistance to ensartinib may limit its therapeutic application in cancer. In this work, we investigated the interaction of ensartinib with P-glycoprotein (P-gp) and ABCG2, two ATP-binding cassette (ABC) multidrug efflux transporters that are commonly associated with the development of multidrug resistance in cancer cells. Our results revealed that P-gp overexpression, but not expression of ABCG2, was associated with reduced cancer cell susceptibility to ensartinib. P-gp directly decreased the intracellular accumulation of ensartinib, and consequently reduced apoptosis and cytotoxicity induced by this drug. The cytotoxicity of ensartinib could be significantly reversed by treatment with the P-gp inhibitor tariquidar. In conclusion, we report that ensartinib is a substrate of P-gp, and provide evidence that this transporter plays a role in the development of ensartinib resistance. Further investigation is needed.

## 1. Introduction

Anaplastic lymphoma kinase (ALK) gene rearrangements that lead to constitutive activation of the ALK kinase are observed in approximately 5% of non–small-cell lung cancer (NSCLC) [1]. Therefore, ALK-targeted therapies are currently used in clinical practices to treat ALK-positive NSCLC patients. Ensartinib (X-396) is a potent second-generation tyrosine kinase inhibitor (TKI) of ALK [2,3]. Preclinical studies have found superior efficacy of ensartinib compared to crizotinib, ceritinib, and alectinib, which are ALK inhibitors that have been approved by the U.S. Food and Drug Administration (FDA) [4]. Clinical studies with ensartinib have been conducted to determine the pharmacokinetics of ensartinib (ClinicalTrials.gov Identifiers: NCT03804541, NCT03510611, NCT03536481, NCT03608007) and its efficacy in patients with ALK-positive NSCLC (NCT02959619, NCT04415320, NCT03753685, NCT02767804, NCT04146571, NCT03215693, NCT03737994), patients with melanoma (NCT03420508), and patients with relapsed or refractory advanced solid tumors, non-Hodgkin lymphoma or histiocytic disorders (NCT03213652, NCT03155620). Clinical studies of combination therapy with ensartinib and durvalumab in ALK-rearranged NSCLC patients (NCT02898116), and combination therapy with ensartinib, carboplatin, pemetrexed and bevacizumab for the treatment of late-stage ALK-positive NSCLC patients (NCT04837716) are also ongoing. Promising results from the first-in-human phase I/II clinical trial of ensartinib (NCT01625234) demonstrated that it is effective and generally well-tolerated in patients with ALK-positive NSCLC [5]. Despite promising results in preclinical and clinical studies, the inevitable emergence of resistance to ensartinib may present a therapeutic problem in the future. For that reason, it is important to investigate the potential mechanism of resistance to ensartinib and find the most appropriate therapeutic strategy to extend its clinical use.

P-glycoprotein (P-gp; ABCB1) and ABCG2 (BCRP; MXR) are members of the ATP-binding cassette (ABC) drug transporter family that are characteristically known for utilizing energy derived from ATP hydrolysis to actively efflux a wide range of cytotoxic and molecularly targeted anticancer agents out of cancer cells [6,7,8,9,10]. Therefore, high expression of P-gp and/or ABCG2 is frequently associated with the development of multidrug resistance (MDR) [10,11,12] and poor prognosis [13,14,15,16,17,18] in patients with several types of blood cancers [13,14,15,16,17,19,20,21,22,23] and solid tumors [18,24,25,26]. Moreover, since P-gp and ABCG2 are also highly expressed at blood-tissue barriers, such as the intestinal epithelium, blood–placenta barrier (BPB), blood-testis barrier (BTB), and the blood–brain barrier (BBB) [10,27,28], the oral absorption and tissue distribution of substrate drugs are significantly affected by these transporters [10,28,29].

In the present study, we investigated the interaction between ensartinib and two main ABC drug transporters (P-gp and ABCG2) to determine the potential impact of these multidrug efflux transporters on the susceptibility of human cancer cells to ensartinib. Our data indicated that ensartinib is a substrate for P-gp, but not for ABCG2, and the intracellular concentration of ensartinib was significantly reduced by the activity of P-gp in human cancer cells. Consequently, P-gp-overexpressing drug-resistant cancer cells were less susceptible to ensartinib than drug-sensitive parental cancer cells. In addition, ensartinib was found to inhibit P-gp-mediated efflux of other substrate drugs, but only at high concentrations. Taken together, our results suggest that the overexpression of P-gp may contribute to increased resistance to ensartinib in tumors. Further investigation is warranted.

## 2. Materials and Methods

### 2.1. Chemicals and Reagents

A Tools Cell Counting (CCK-8) kit was acquired from Biotools Co., Ltd. (Taipei, Taiwan). An Annexin V FITC-Apoptosis Detection Kit was acquired from BD Pharmingen (San Diego, CA, USA). The ALK inhibitor ensartinib (X-396) was acquired from Selleckchem (Houston, TX, USA). Dulbecco’s Modified Eagle’s medium (DMEM), Roswell Park Memorial Institute medium 1640 (RPMI-1640), and fetal calf serum (FCS) were acquired from Gibco/Thermo Fisher Scientific, Inc. (Waltham, MA, USA). All the chemicals used for this study were obtained from Sigma-Aldrich (St. Louis, MO, USA) unless stated otherwise.

### 2.2. Cell Culture

The KB-3-1 and KB-V1 cell lines [30], the OVCAR-8 and NCI-ADR-RES [31], and human embryonic kidney (HEK293) cells stably transfected with either empty pcDNA 3.1 vector (pcDNA 3.1-HEK293), human P-gp (MDR19-HEK293) [32] or human ABCG2 (R482-HEK293) [33] were cultured in DMEM medium. The H460 and H460-MX20 cell lines [34] and the S1 and S1-MI-80 cell lines [35] were cultured in RPMI-1640 medium. The KB-V1 cell line [36], the NCI-ADR-RES cell line [31], HEK293-transfected lines [37], the H460-MX20 cell line [38], and the S1-MI-80 cell line [35] were cultured with the addition of vinblastine (1 mg/mL), doxorubicin (0.85 μM), G418 (2 mg/mL), and mitoxantrone at 20 nM or 80 μM, respectively. Cell lines were generous gifts from Drs. Michael Gottesman and Susan Bates (NCI, NIH, Bethesda, MD, USA). All cell lines were cultured in medium supplemented with 10% FCS, 2 mM L-glutamine and 100 units of penicillin/streptomycin/mL at 37 °C in 5% CO_2_ humidified air and placed in drug-free medium 7 days before assay.

### 2.3. Cytotoxicity Assays

Cytotoxicity assays were performed as described previously [39]. Briefly, cells were seeded in 96-well flat-bottom plates at a density of 5000 cells per well in drug-free DMEM medium or RPMI 1640 medium supplemented with 10% FCS, 2 mM L-glutamine and incubated overnight at 37 °C in 5% CO_2_ humidified air. Cells were then treated with ensartinib or other compounds with 0.5% (v/v) final concentration of DMSO in each well for an additional 72 h, and subsequently processed using CCK-8 or MTT reagents as previously described [40].

### 2.4. Western Blots

Western blot analysis of P-gp protein was performed as described previously [41]. The primary antibodies C219 at 1:3000 (#517310, Merck Millipore, Burlington, MA, USA), and anti-alpha-tubulin at 1:100,000 dilution (#T6199, Sigma-Aldrich, St. Louis, MO, USA) were used to identify P-gp, with tubulin as the positive loading control. Horseradish peroxidase-conjugated goat anti-mouse immunoglobulin G (IgG) 1:100000 dilution (Abcam, Cambridge, MA, USA) was used as the secondary antibody. Signals were detected as previously described [41].

### 2.5. Fluorescent Calcein and Pheophorbide A Accumulation Assay

The accumulation of P-gp substrate calcein-AM and the ABCG2 substrate pheophorbide A (PhA) was determined using flow cytometry analysis as described previously [39]. Briefly, trypsinized cells were incubated in IMDM containing 5% FCS with calcein-AM or PhA with the addition of DMSO (control), 10 μM ensartinib, or a reference inhibitor for P-gp (5 μM tariquidar) or ABCG2 (5 μM Ko143), and the relative fluorescence intensity of calcein or PhA was analyzed as previously described [42].

### 2.6. Ultra-Performance Liquid Chromatography (UPLC)-Selected Reaction Monitoring Mass Spectrometry (SRM/MS)-Based Drug Accumulation Assay

The intracellular ensartinib was extracted and semi-quantified according to the methods reported in our previous studies [39,43]. Briefly, 2 × 10^6^ cells were incubated with 10 μM ensartinib in the absence or presence of 10 μM tariquidar at 37 °C for 60 min and processed as described previously [39,43]. Mobile phase A: 0.1% formic acid in water. Mobile phase B: 0.1% formic acid in acetonitrile. The gradient method used was: (t = 0 min, 10% B; t = 0.5 min, 10% B; t = 4.0 min, 35% B; t = 6.0 min, 60% B; t = 6.5 min, 90% B; t = 8.2 min, 90% B; t = 8.5 min, 10% B), followed by equilibration for 3.5 min (60 µL/min flow rate with a constant column temperature of 40 °C). The analysis was performed using the Waters ACQUITY UPLC system with a Waters BEH C18 Column (130 Å, 1.7 μm, 1 × 100 mm) coupled with HCT ultra (Bruker Daltonik GmbH, Bremen, Germany) by SRM in positive mode. The SRM transition of ensartinib precursor ion 561 (mass-to charge m/z) was integrated using DataAnalysis 4.2 software (Bruker Corporation, Billerica, MA, USA), and the quantitative fragment ion m/z 371 was calculated by peak area. The standard was prepared from ensartinib stock by serial dilution, and an equal matrix background of the cell lysates without ensartinib treatment was added to construct respective calibration curves for LC-SRM/MS analysis. These calibration curves were subsequently used to semi-quantify the intracellular accumulation of ensartinib in KB cells after ensartinib treatment in the presence or absence of tariquidar. The standard response curves of ensartinib were generated by analyzing four injections at each of the seven concentration levels under the same operating conditions using cell lysate extracts as background, ranging from 62.5 fmol/μL to 4 pmol/μL. The concentration of the ensartinib response curve was set, ranging from 62.5 fmol/μL to 4 pmol/μL, using the extracts of cell lysate as a background.

### 2.7. Apoptosis Assays

The annexin V–FITC and propidium iodide (PI) staining method for the detection of apoptosis was performed as reported by Anderson et al. [44]. Briefly, cells were treated either with DMSO (control), ensartinib (2 μM), or tariquidar (1 μM) or ensartinib in combination with tariquidar for 48 h before being processed and analyzed using a FACSort flow cytometer (Becton-Dickinson Biosciences, San Jose, CA, USA) equipped with CellQuest software as described previously [41].

### 2.8. Docking of Ensartinib in the Substrate-Binding Pocket of P-gp

The energy was minimized for the cryo-EM structure of the inward-open conformation of P-gp (PDBID:6QEX) [45] and ensartinib with a CHARMM force field at pH 7.4 in the Accelrys Discovery Studio 4.0 as previously described [46]. The MGLtools software package (Scripps Research Institute) was used to prepare the structures of P-gp and ensartinib [47]. The Pymol molecular graphics system, Version 1.7 (Schrödinger, LLC, New York, NY, USA) was used to analyze the docked poses.

### 2.9. Data Analysis

Curve fitting was performed in GraphPad Prism (GraphPad Software 3.0, La Jolla, CA, USA). Statistical data analysis (two-sided Student’s *t*-test) was carried out using KaleidaGraph (Synergy Software, Reading, PA, USA) software. The values were mean ± standard deviation (SD) or mean ± standard error of the mean (SEM), calculated from at least three independent experiments. The difference between mean values of experimental and control or improvement in fit was considered as “statistically significant” if the probability, *p*, was less than 0.05, and labeled with asterisks.

## 3. Results

### 3.1. P-gp Confers Resistance to Ensartinib

**Figure 1:**
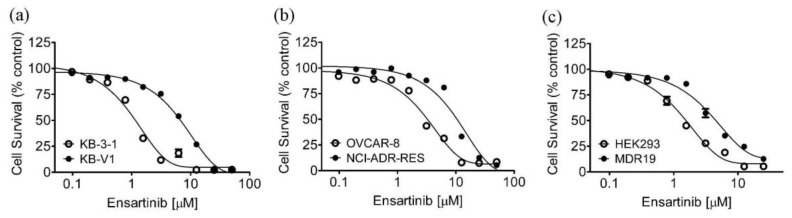
The antiproliferative effect of ensartinib is reduced in cells overexpressing P-glycoprotein. The cytotoxicity of ensartinib in (**a**) KB-3-1 (open circles) and the P-gp-overexpressing subline KB-V1 (filled circles); (**b**) OVCAR-8 (open circles) and the P-gp-overexpressing subline NCI-ADR-RES (filled circles); as well as (**c**) pcDNA3.1-HEK293 (open circles) and the P-gp-transfected MDR19-HEK293 (filled circles), was determined as described previously [39]. The values were mean ± SEM calculated from at least three independent experiments.

To determine if P-gp or ABCG2 expression would confer resistance to ensartinib, cytotoxicity assays were performed on several pairs of drug-sensitive cancer cell lines and the corresponding drug-resistant cell lines overexpressing either P-gp or ABCG2. Cytotoxicity assays revealed that the P-gp-overexpressing human KB-V1 epidermal cancer cell line was six-fold more resistant to ensartinib (Figure 1a), and the P-gp-overexpressing human NCI-ADR-RES ovarian cancer cell line was three-fold more resistant to ensartinib (Figure 1b) as compared to the parental KB-3-1 and OVCAR-8 cancer cell lines. In contrast, ensartinib was equally cytotoxic to the ABCG2-overexpressing human S1-MI-80 colon cancer cells and the ABCG2-overexpressing H460-MX20 human non-small-cell lung cancer (NSCLC) cells as to the parental S1 and NCI-H460 cancer cells (Table 1). The cytotoxicity of ensartinib was also examined using HEK293 cells transfected with empty vector (pcDNA3.1-HEK293), P-gp (MDR19-HEK293) or ABCG2 (R482-HEK293). The fact that the MDR19-HEK293 cell line was three-fold more resistant to ensartinib (Figure 1c) is in agreement with our observation concerning P-gp-overexpressing cancer cell lines. Since our data suggested that ensartinib resistance was mediated by P-gp, the cytotoxicity of ensartinib was determined with or without 1 μM tariquidar, a P-gp inhibitor. As shown in Table 1, tariquidar significantly reversed resistance to ensartinib in P-gp-overexpressing cell lines without affecting the parental cell lines, indicating that ensartinib was definitely a substrate for P-gp.

### 3.2. Ensartinib Attenuates the Drug Efflux Function of P-gp

**Figure 2:**
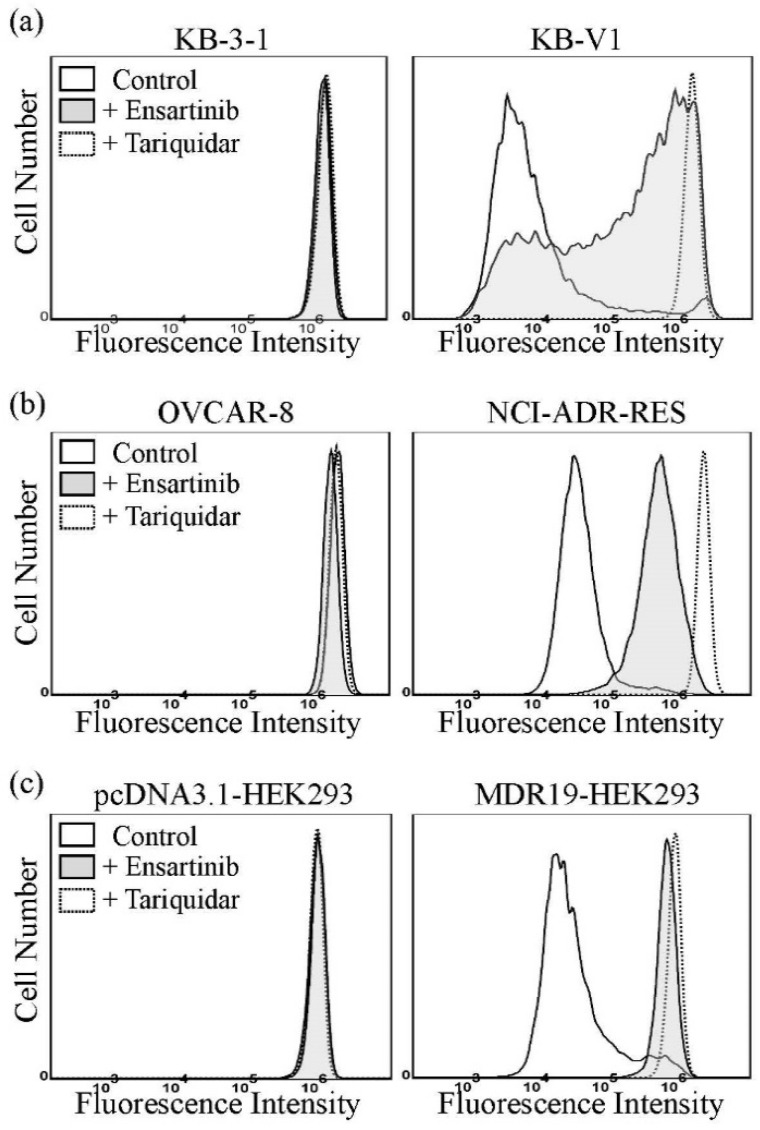

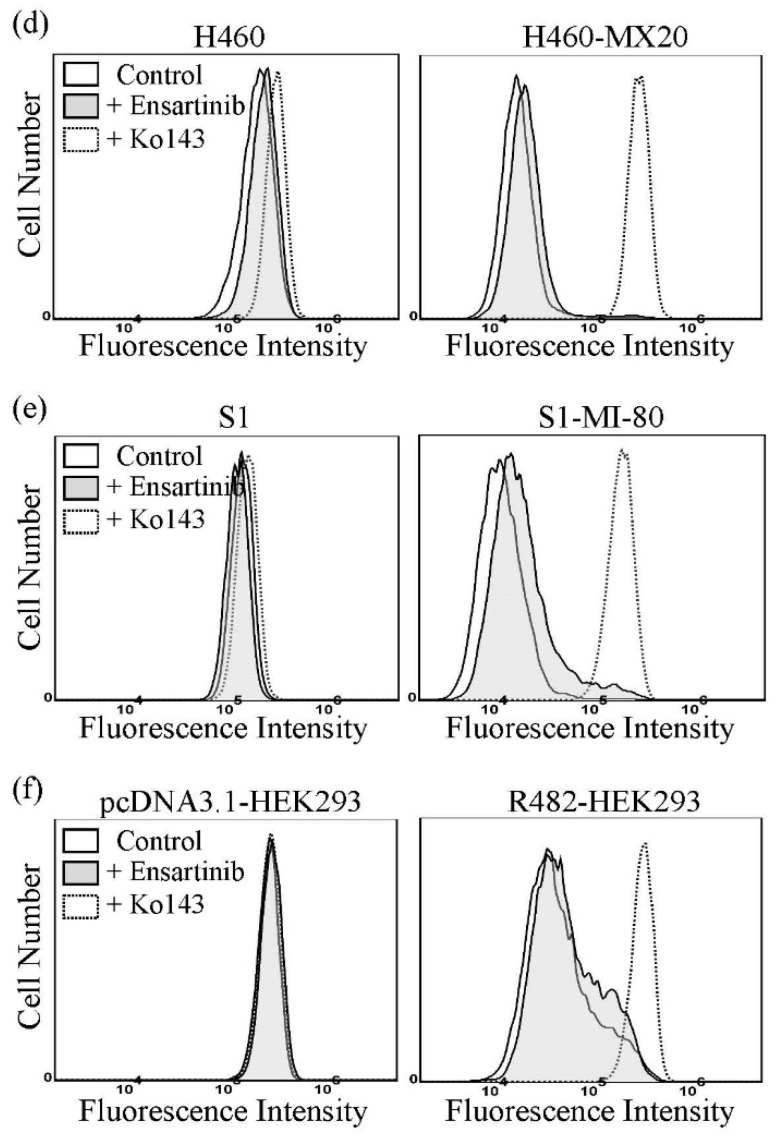
Ensartinib increases the intracellular accumulation of calcein in P-glycoprotein-overexpressing cells. The effect of ensartinib on the accumulation of the P-gp substrate drug calcein-AM (**a**–**c**) or accumulation of the ABCG2 substrate pheophorbide A (**d**–**f**) was determined in (**a**) KB-3-1 cells (left panel) and P-gp-overexpressing KB-V1 cells (right panel), (**b**) OVCAR-8 cells (left panel) and P-gp-overexpressing NCI-ADR-RES cells (right panel), (**c**) pcDNA3.1-HEK293 (left panel) and P-gp-transfected MDR19-HEK293 (right panel) cells, (**d**) NCI-H460 (left panel) and ABCG2-overexpressing H460-MX20 cells, (**e**) S1 (left panel) and ABCG2-overexpressing S1-MI-80 cells (right panel), and (**f**) pcDNA3.1-HEK293 (left panel) and ABCG2-transfected R482-HEK293 cells (right panel). Cells were treated with DMSO (control, solid lines), 10 μM ensartinib (filled solid lines), or 5 μM tariquidar (**a**–**c**, dotted lines) as a positive control for P-gp or 5 μM Ko143 (**d**–**f**, dotted lines) as a positive control for ABCG2. Cells were processed and analyzed as described in Materials and Methods. Representative histograms from at least three independent experiments are shown.

Previous studies have demonstrated that, at higher concentrations, certain substrates of P-gp and/or ABCG2 can act as inhibitors of P-gp and/or ABCG2, respectively [37,48,49,50]. To this end, the ability of ensartinib to inhibit P-gp-mediated calcein-AM efflux and ABCG2-mediated PhA efflux was examined. As shown in Figure 2, the accumulation of calcein, a fluorescent product of the P-gp substrate calcein-AM [51], in KB-V1 (Figure 2a), NCI-ADR-RES (Figure 2b), and MDR19-HEK293 (Figure 2c) cells was extremely low (dotted lines). However, the intracellular fluorescence level increased significantly when these cells were incubated with calcein-AM in the presence of 10 μM ensartinib (filled solid lines), or 5 μM tariquidar (dotted lines). In contrast, the accumulation of the fluorescent ABCG2 substrate PhA [42] in H460-MX20 (Figure 2d), S1-MI-80 (Figure 2e), and R482-HEK293 (Figure 2f) cells increased substantially when these cells were incubated with PhA in the presence of 5 μM Ko143, a positive control (dotted lines), but remained low in the presence of 10 μM ensartinib (filled solid lines). These results suggest that at higher concentrations, ensartinib could compete with the transport of other substrates mediated by P-gp.

Next, the effect of ensartinib on reversing MDR mediated by P-gp and ABCG2 was determined in P-gp- and ABCG2-overexpressing multidrug-resistant cells. The extent of reversal was determined by adding a non-toxic concentration of ensartinib, tariquidar or Ko143 to the cytotoxicity assays, and presented as a fold-reversal (FR) value as described previously [48,52]. Reversal assays revealed that, at the highest non-toxic concentration of 500 nM, ensartinib had no significant effect on P-gp-mediated resistance to the P-gp substrates paclitaxel, vincristine or colchicine [53] in P-gp-overexpressing KB-V1 and NCI-ADR-RES cancer cell lines, and the P-gp-transfected MDR19-HEK293 cell line (Table 2). Similarly, ABCG2-mediated resistance to the ABCG2 drug substrates mitoxantrone, topotecan and SN-38 [54,55] was not reversed by ensartinib in ABCG2-overexpressing S1-MI-80 or H460-MX20 cancer cell lines, or in the ABCG2-transfected R482-HEK293 cell line (Table 3). Our results revealed that, although ensartinib could block P-gp-mediated drug efflux at higher concentrations, it could not resensitize P-gp- or ABCG2-overexpressing cancer cells to cytotoxic drugs at non-toxic low concentrations.

### 3.3. P-gp Reduces the Intracellular Accumulation of Ensartinib in Human Cancer Cells

**Figure 3:**
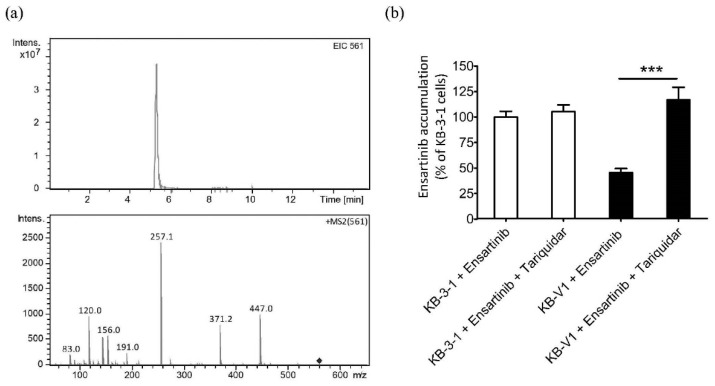
P-gp-mediated transport decreases the intracellular concentration of ensartinib in human cancer cells. (**a**) The chemical structure (precursor ion m/z 561 in positive mode) and the mass spectra of major fragment ions of ensartinib. The fragment ion m/z 371 was selected for quantitative analysis. (**b**) The intracellular accumulation of ensartinib in KB-3-1 (white bars) and KB-V1 cells (black bars) treated with 10 μM of ensartinib in the absence or presence of tariquidar was determined as described in Materials and Methods. Quantitative data are presented as mean values ± S.D. calculated from three independent experiments. *** *p* < 0.001, as compared to treatment with tariquidar.

Knowing that P-gp expression confers resistance to ensartinib, and that one of the most likely explanations for this is the reduced intracellular drug accumulation caused by P-gp-mediated drug efflux [10], we examined the effect of P-gp function on the intracellular accumulation of ensartinib in cancer cells. KB-3-1 and the P-gp-overexpressing variant KB-V1 were treated with 10 μM ensartinib in the absence or presence of 10 μM tariquidar, and the intracellular concentration of ensartinib was determined using a liquid chromatography-selected reaction monitoring mass spectrometry (LC-SRM/MS) method (Figure 3a) as described previously [39,41,43]. Human KB epidermal cancer cell lines were chosen for this study since KB-V1 showed the highest RF value among all the P-gp-overexpressing cell lines (Table 1). As shown in Figure 3b, significantly lower intracellular accumulation of ensartinib was detected in KB-V1 cancer cells (filled bars) compared to KB-3-1 cancer cells (open bars) or cells treated with ensartinib in the presence of tariquidar.

**Figure 4:**
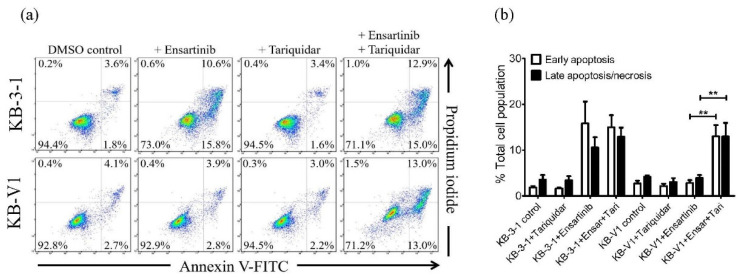
Ensartinib induces apoptosis in cancer cell lines. (**a**) Representative dot plots of the extent of apoptosis determined in KB-3-1 cells (upper panels) and the P-gp-overexpressing subline KB-V1 (lower panels) treated with DMSO (DMSO control), 2 µM ensartinib (+ Ensartinib), 1 µM tariquidar (+ Tariquidar), or a combination of 2 µM ensartinib and 1 µM tariquidar (+ Ensartinib + Tariquidar) for 48 h. Cells were processed and analyzed as described in Materials and Methods and as described previously [40]. (**b**) Quantitative data are presented as mean values ± S.D. obtained from at least three independent experiments. ** *p* < 0.01, versus the same treatment in the presence of tariquidar.

The effect of tariquidar on ensartinib-induced apoptosis was also examined. As shown in Figure 4, without inducing apoptosis in KB-V1 cells, the apoptotic KB-3-1 cell population was substantially increased by ensartinib, from an approximate 5% basal level to 26%. More importantly, the ensartinib-induced KB-V1 apoptotic cell population was significantly increased by tariquidar to a level comparable to that observed in KB-3-1 cells. Of note, tariquidar alone had no significant effect on the apoptotic cell population in either KB-3-1 or KB-V1 cell lines. These results were in agreement with the cytotoxicity data (Table 1), suggesting that P-gp mediates the efflux of ensartinib and contributes to the reduced efficacy of ensartinib in P-gp-overexpressing cancer cells.

### 3.4. Docking of Ensartinib in the Drug-Binding Pocket of P-gp

**Figure 5:**
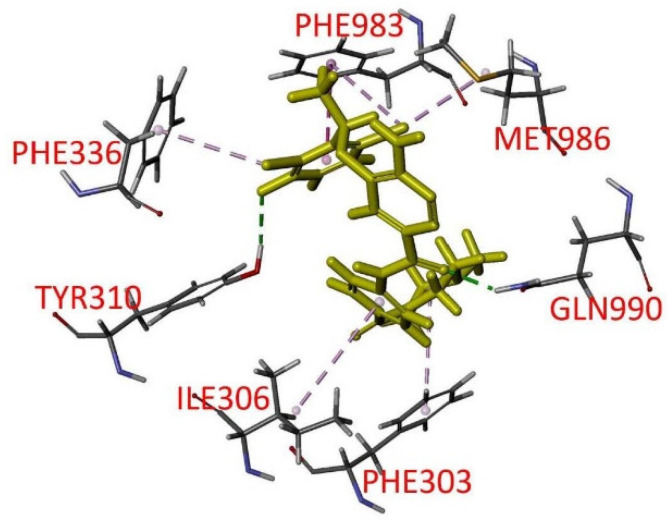
Docking of ensartinib to P-gp. The lowest energy binding mode of ensartinib with Taxol-bound human P-gp (PDB: 6QEX) was predicted by Accelrys Discovery Studio 4.0 software as described in Materials and Methods. The molecular model of ensartinib is highlighted in yellow, and the atoms for interacting amino acid residues carbon, hydrogen, nitrogen, and oxygen are colored gray, light gray, blue, and red, respectively. Dotted lines indicate proposed interactions.

To further investigate the interactions between ensartinib and the substrate-binding pocket of P-gp, the in silico molecular docking analysis of ensartinib in the inward-open structure of P-gp (PDBID:6QEX) [45] was carried out. The lowest energy docking pose revealed a similar binding location for ensartinib as reported for the Taxol- and vincristine-bound cryo-EM structures. Ensartinib was shown to interact with numerous hydrophobic and aromatic residues in the transmembrane domains (TMDs) such as PHE303, ILE306, TYR310, PHE336, PHE983, MET986, and a hydrogen bond was predicted between its carbonyl group and GLN990 (Figure 5). Common interacting residues were also observed in the binding with Taxol (PHE983, MET986 and GLN990) and vincristine (ILE306, TYR310, PHE983, MET986 and GLN990) [45,56].

## 4. Discussion

Despite the tremendous progress made in recent years in the development of novel molecularly-targeted therapeutic agents, resistance often occurs within months, caused by resistance mechanisms including the activation of bypass tracks, secondary mutations, and amplification of resistance genes [3,56]. Nonetheless, the understanding of molecular mechanisms underlying resistance to these molecularly targeted therapeutic agents remains incomplete, which poses a therapeutic challenge. Previous studies have demonstrated that the drug efflux function of P-gp and ABCG2 could alter the bioavailability, distribution and efficacy of epidermal growth factor receptor (EGFR) inhibitors [57,58,59], vascular endothelial growth factor receptor (VEGFR) inhibitors [60], rapidly accelerated fibrosarcoma (RAF) inhibitors [61,62,63,64], PARP inhibitors [65,66,67], and ALK inhibitors [68,69,70,71]. For example, Tang and colleagues reported that the first-generation ALK inhibitor crizotinib is a good transport substrate of P-gp, but not of ABCG2 [68]. In contrast, the second-generation ALK inhibitors ceritinib [69] and brigatinib [71] are both transport substrates of P-gp and ABCG2. Moreover, Katayama and colleagues revealed that P-gp mediates resistance to both crizotinib and ceritinib in NSCLC patients [70]. In this study, we sought to determine the potential impact of P-gp and ABCG2 on the susceptibility of human cancer cells to the second-generation ALK inhibitor ensartinib.

Ensartinib is a specific ALK TKI that is cytotoxic against a wide variety of cancer cell lines, including crizotinib-resistant cancer cell lines known to harbor ALK fusions or point mutations. Other than the HepG2 hepatocellular cancel cell line, the reported IC_50_ values of ensartinib against the cancer cell lines tested were in the range of 15 nM to 3.0 μM [2]. In our study, the IC_50_ values of ensartinib for drug-sensitive cancer cell lines ranged from 0.9 μM to 3.0 μM, comparable to the IC_50_ values reported in previous studies [2,72]. On the other hand, we observed that the IC_50_ values of ensartinib for P-gp-overexpressing cancer cell lines, regardless of the tissue of origin, and HEK293 cells with ectopic expression of P-gp were significantly higher than the IC_50_ values in the respective drug-sensitive parental cells, which could be reversed by the P-gp inhibitor tariquidar (Table 1). Interestingly, Lovly and colleagues found that ensartinib exhibited a significantly higher IC_50_ value for the HepG2 cancer cell line as compared to other cell lines [2], which may be due, at least partially, to the activity of P-gp presented in the HepG2 cancer cells [73,74,75]. In addition, we discovered ensartinib could inhibit the P-gp-mediated efflux of the P-gp substrate calcein-AM from P-gp-expressing cells but had no significant effect on ABCG2-mediated efflux of its substrate PhA (Figure 2). The results of lower intracellular ensartinib accumulation (Figure 3) and reduced ensartinib-induced apoptosis (Figure 4) in P-gp-overexpressing KB-V1 cancer cells compared to the parental KB-3-1 cells correspond directly to the cytotoxicity data. Moreover, by blocking the drug-efflux function of P-gp, tariquidar was able to restore the intracellular concentration of ensartinib and ensartinib-induced apoptosis in KB-V1 cells, which is in agreement with our conclusion that P-gp reduces the efficacy of ensartinib in human cancer cells. Analysis of in silico molecular docking of ensartinib with the inward-open structure of P-gp (PDBID:6QEX) [45] revealed several common residues that interact with Taxol, vincristine and ensartinib. The docking analysis further provides support for binding of ensartinib in the drug-binding pocket that is similar to that of Taxol or vincristine (Figure 5). Collectively, these data indicate that P-gp mediates ensartinib resistance in multidrug-resistant cancer cells.

It is worth noting that Vagiannis and colleagues reported previously that ensartinib is an effective inhibitor of P-gp and ABCG2 but behaved as a substrate of P-gp in Madin-Darby canine kidney II (MDCKII) monolayer transport assays, and that ensartinib stimulated P-gp-specific ATPase activity in a concentration-dependent manner [76]. Consistent with our results, these authors found that ensartinib is not transported by ABCG2 in MDCK-II cells. However, they found that the antitumor efficiency of ensartinib was not compromised by P-gp in MDCKII, HL60, or A431 cells, but with IC_50_ values that were considerably higher than the IC_50_ values reported in other studies [2,72,76,77]. The discrepancy between results with MDCK-II and human cell lines used in our study could be due to the presence of endogenous canine P-gp and ABCG2 transporters. Another difference is that for the docking studies, Vagiannis et al. used a homology model of human P-gp based on the mouse Abcb1a structure. In addition, they docked ensartinib in the NBDs of the ATP-bound structure of the EQ mutant of human P-gp (pdb.6C0V; Figure 5). Using this structure is not logical, as the drug-binding pocket is collapsed and there is no evidence for binding of ensartinib or any other structure in the NBDs [76]. In the same study, they reported that ensartinib at 10 μM could reverse P-gp-mediated resistance to daunorubicin and ABCG2-mediated resistance to mitoxantrone in MDCKII cells transduced with P-gp or ABCG2, and in the human leukemia HL60 cell line overexpressing P-gp or ABCG2. However, considering that ensartinib at 10 μM is cytotoxic to parental MDCKII and HL60 cell lines, the chemosensitizing effect of ensartinib may be overestimated [76]. It is worth noting that, in our study, we found that at the highest non-toxic concentration of 0.5 μM, ensartinib was unable to reverse P-gp-mediated resistance to the P-gp substrates paclitaxel, vincristine, and colchicine, or ABCG2-mediated resistance to the ABCG2 substrates mitoxantrone, SN-38, and topotecan.

## 5. Conclusions

In summary, although experimental results from cell or animal models of multidrug-resistant cancers do not necessarily reflect clinical reality [78], our data reveal that ensartinib is a substrate for P-gp. Based on our findings, we propose that P-gp expression could contribute to the development of resistance to ensartinib resistance in cancer cells and have clinical implications in NSCLC patients receiving this drug treatment. In addition, the activity of P-gp could lead to altered pharmacokinetics and pharmacodynamics in certain patients and should be further investigated.

## Figures and Tables

**Table 1:** Cytotoxicity of ensartinib in drug-sensitive and multidrug-resistant cells overexpressing either P-glycoprotein or ABCG2.

Cell Line	Type	Transporter Expressed	IC_50_ ± SD [μM] ^1^	
			Ensartinib	Ensartinib + Tariquidar
KB-3-1	Epidermal cancer	-	0.92 ± 0.19	0.77 ± 0.14
KB-V1	Epidermal cancer	P-gp	5.86 ± 1.17 **	0.88 ± 0.14 **
OVCAR-8	Ovarian cancer	-	3.00 ± 0.57	3.01 ± 0.59
NCI-ADR-RES	Ovarian cancer	P-gp	9.20 ± 2.42 *	5.57 ± 0.93 *
S1	Colon cancer	-	2.84 ± 0.33	NA
S1-MI-80	Colon cancer	ABCG2	3.69 ± 0.63	NA
NCI-H460	NSCLC	-	1.75 ± 0.41	NA
H460-MX20	NSCLC	ABCG2	2.45 ± 0.51	NA
pcDNA3.1-HEK293	-	-	1.30 ± 0.14	1.12 ± 0.09
MDR19-HEK293	-	P-gp	4.42 ± 0.66 **	1.94 ± 0.23 **
R482-HEK293	-	ABCG2	1.80 ± 0.28	NA

Abbreviation: NA, not applicable. ^1^ IC_50_ values are mean ± SD calculated from at least three independent experiments as described in Materials and Methods. * *p* < 0.05; ** *p* < 0.01.

**Table 2:** Effect of ensartinib on reversing P-gp-mediated multidrug resistance in drug-resistant human cell lines.

Compounds	Concentration (nM)	IC_50_ ^1^ ± SD and (FR ^2^)	
		KB-3-1 (Parental) [nM]	KB-V1 (ABCB1) [μM]
Paclitaxel	-	2.45 ± 0.55 (1.0)	7.08 ± 1.17 (1.0)
+ ensartinib	500	2.90 ± 0.70 (0.8)	7.18 ± 1.19 (1.0)
+ tariquidar	1000	3.01 ± 0.80 (0.8)	2.41 ± 0.61 [nM] *** (2938)
Vincristine	-	2.18 ± 0.48 (1.0)	3.25 ± 0.46 (1.0)
+ ensartinib	500	2.94 ± 0.62 (0.7)	3.43 ± 0.29 (0.9)
+ tariquidar	1000	1.76 ± 0.40 (1.2)	4.00 ± 0.81 [nM] *** (813)
Colchicine	-	15.87 ± 5.22 (1.0)	1.04 ± 0.05 (1.0)
+ ensartinib	500	19.85 ± 6.47 (0.8)	1.38 ± 0.26 (0.8)
+ tariquidar	1000	14.65 ± 4.87 (1.1)	16.31 ± 4.19 [nM] *** (64)
		OVCAR-8 (Parental) [nM]	NCI-ADR-RES (ABCB1) [μM]
Paclitaxel	-	5.10 ± 1.10 (1.0)	9.81 ± 1.79 (1.0)
+ ensartinib	500	4.32 ± 0.95 (1.2)	10.03 ± 1.71 (1.0)
+ tariquidar	1000	4.30 ± 1.04 (1.2)	7.67 ± 0.73 [nM] *** (1279)
Vincristine	-	9.38 ± 1.41 (1.0)	5.74 ± 0.88 (1.0)
+ ensartinib	500	7.53 ± 1.17 (1.2)	5.79 ± 0.82 (1.0)
+ tariquidar	1000	6.74 ± 1.21 (1.4)	28.56 ± 3.24 [nM] *** (201)
Colchicine	-	26.32 ± 7.57 (1.0)	2.14 ± 0.45 (1.0)
+ ensartinib	500	28.26 ± 8.94 (0.9)	2.33 ± 0.48 (0.9)
+ tariquidar	1000	24.07 ± 7.29 (1.1)	45.70 ± 13.54 [nM] ** (47)
		pcDNA3.1-HEK293 (Parental) [nM]	MDR19-HEK293 (ABCB1) [nM]
Paclitaxel	-	2.10 ± 0.36 (1.0)	1583.40 ± 212.71 (1.0)
+ ensartinib	500	1.73 ± 0.40 (1.2)	1385.32 ± 174.42 (1.1)
+ tariquidar	1000	2.28 ± 0.42 (0.9)	3.49 ± 0.61 *** (453.70)
Vincristine	-	2.75 ± 0.25 (1.0)	794.92 ± 123.55 (1.0)
+ ensartinib	500	3.33 ± 0.47 (0.8)	769.60 ± 159.32 (1.0)
+ tariquidar	1000	2.89 ± 0.44 (1.0)	1.44 ± 0.29 ** (552.03)
Colchicine	-	12.76 ± 3.28 (1.0)	195.23 ± 36.89 (1.0)
+ ensartinib	500	14.54 ± 2.94 (0.9)	235.53 ± 66.08 (0.8)
+ tariquidar	1000	12.66 ± 2.98 (1.0)	7.69 ± 1.44 *** (25.39)

Abbreviation: FR, fold-reversal. ^1^ IC_50_ values are mean ± SD calculated from at least three independent experiments. ^2^ FR values were calculated by dividing the IC_50_ value of a known P-gp substrate drug by the IC_50_ value of the same substrate drug in the presence of ensartinib or tariquidar. ** *p* < 0.01; *** *p* < 0.001.

**Table 3:** Effect of ensartinib on reversing ABCG2-mediated multidrug resistance in drug-resistant human cell lines.

Compounds	Concentration (nM)	IC_50_ ^1^ ± SD and (FR ^2^)	
		S1 (parental) [nM]	S1-MI-80 (ABCG2) [μM]
Mitoxantrone	-	9.52 ± 2.63 (1.0)	31.41 ± 6.49 (1.0)
+ ensartinib	500	7.08 ± 2.20 (1.3)	27.51 ± 5.32 (1.1)
+ Ko143	1000	7.52 ± 2.11 (1.3)	0.94 ± 0.15 ** (33.4)
		[nM]	[μM]
Topotecan	-	57.09 ± 10.95 (1.0)	33.20 ± 3.08 (1.0)
+ ensartinib	500	58.00 ± 9.59 (1.0)	27.19 ± 3.25 (1.2)
+ Ko143	1000	60.77 ± 11.40 (0.9)	1.69 ± 0.32 *** (19.6)
		[nM]	[μM]
SN-38	-	13.02 ± 2.67 (1.0)	5.45 ± 1.25 (1.0)
+ ensartinib	500	11.97 ± 2.26 (1.1)	9.04 ± 1.93 (0.6)
+ Ko143	1000	12.56 ± 2.74 (1.0)	0.12 ± 0.04 * (45.4)
		NCI-H460 (parental) [nM]	H460-MX20 (ABCG2) [μM]
Mitoxantrone	-	70.76 ± 10.55 (1.0)	1.07 ± 0.15 (1.0)
+ ensartinib	500	50.67 ± 10.42 (1.4)	0.94 ± 0.19 (1.1)
+ Ko143	1000	35.22 ± 7.44 ** (2.0)	0.12 ± 0.03 *** (8.9)
		[nM]	[nM]
Topotecan	-	105.58 ± 12.64 (1.0)	799.88 ± 173.21 (1.0)
+ ensartinib	500	111.29 ± 17.03 (0.9)	775.68 ± 146.16 (1.0)
+ Ko143	1000	46.63 ± 6.32 ** (2.3)	39.09 ± 10.01 ** (20.5)
		[nM]	[nM]
SN-38	-	28.66 ± 3.10 (1.0)	273.60 ± 52.10 (1.0)
+ ensartinib	500	27.42 ± 2.86 (1.0)	247.83 ± 31.09 (1.1)
+ Ko143	1000	8.27 ± 1.76 *** (3.5)	4.23 ± 1.25 *** (64.7)
		pcDNA3.1-HEK293 (parental) [μM]	R482-HEK293 (ABCG2) [μM]
Mitoxantrone	-	4.99 ± 0.61 (1.0)	114.63 ± 13.10 (1.0)
+ ensartinib	500	4.65 ± 0.40 (1.1)	126.89 ± 11.12 (0.9)
+ Ko143	1000	4.54 ± 0.46 (1.1)	9.47 ± 0.86 *** (12.1)
		[nM]	[nM]
Topotecan	-	31.45 ± 5.00 (1.0)	669.76 ± 77.52 (1.0)
+ ensartinib	500	29.76 ± 5.52 (1.1)	810.79 ± 94.79 (0.8)
+ Ko143	1000	33.30 ± 6.35 (0.9)	144.57 ± 27.46 *** (4.6)
		[nM]	[nM]
SN-38	-	4.06 ± 0.76 (1.0)	322.50 ± 33.86 (1.0)
+ ensartinib	500	3.94 ± 0.89 (1.0)	351.15 ± 41.89 (0.9)
+ Ko143	1000	4.00 ± 0.76 (1.0)	15.48 ± 3.51 *** (20.8)

Abbreviation: FR, fold-reversal. ^1^ IC_50_ values are mean ± SD calculated from at least three independent experiments. ^2^ FR values were calculated by dividing the IC_50_ value of a known ABCG2 substrate drug by the IC_50_ value of the same substrate drug in the presence of ensartinib or Ko143. * *p* < 0.05; ** *p* < 0.01; *** *p* < 0.001.

## Data Availability

Not Applicable.
